# Neotropical Frog Foam Nest’s Microbiomes

**DOI:** 10.3390/microorganisms11040900

**Published:** 2023-03-30

**Authors:** Felipe Augusto Correia Monteiro, Saulo Gonçalves de Santiago Bezerra, Luzia Gabrielle Zeferino de Castro, Francisca Andrea da Silva Oliveira, Leonardo Ribeiro Oliveira Normando, Vânia Maria Maciel Melo, Denise Cavalcante Hissa

**Affiliations:** 1 Programa de Pós-Graduação em Biotecnologia—Rede Nordeste de Biotecnologia, Universidade Federal do Ceará, Fortaleza 60020-181, Brazil; felipemonteiroifce@gmail.com (F.A.C.M.); vmmmelo@gmail.com (V.M.M.M.); 2 Instituto Federal de Educação, Ciência e Tecnologia do Ceará, Campus Jaguaribe, Rua Pedro Bezerra de Menezes, Jaguaribe 63475-000, Brazil; 3 Laboratório de Recursos Genéticos, Departamento de Biologia, Universidade Federal do Ceará, Av. Humberto Monte 2977, Campus do Pici, Bloco 909, Fortaleza 60455-760, Brazil; 4 Laboratório de Ecologia Microbiana e Biotecnologia, Departamento de Biologia, Universidade Federal do Ceará, Av. Humberto Monte 2977, Campus do Pici, Bloco 909, Fortaleza 60455-760, Brazil

**Keywords:** leptodactylidae, reproductive modes, biosurfactants, bacteriome, frog defenses

## Abstract

Amphibian foam nests are unique microenvironments that play a crucial role in the development of tadpoles. They contain high levels of proteins and carbohydrates, yet little is known about the impact of their microbiomes on tadpole health. This study provides a first characterization of the microbiome of foam nests from three species of Leptodactylids (*Adenomera hylaedactyla*, *Leptodactylus vastus*, and *Physalaemus cuvieri*) by investigating the DNA extracted from foam nests, adult tissues, soil, and water samples, analyzed via 16S rRNA gene amplicon sequencing to gain insight into the factors driving its composition. The results showed that the dominant phyla were proteobacteria, bacteroidetes, and firmicutes, with the most abundant genera being *Pseudomonas*, *Sphingobacterium*, and *Paenibacillus*. The foam nest microbiomes of *A. hylaedactyla* and *P. cuvieri* were more similar to each other than to that of *L. vastus*, despite their phylogenetic distance. The foam nests demonstrated a distinct microbiome that clustered together and separated from the microbiomes of the environment and adult tissue samples. This suggests that the peculiar foam nest composition shapes its microbiome, rather than vertical or horizontal transference forces. We expanded this knowledge into amphibian foam nest microbiomes, highlighting the importance of preserving healthy foam nests for amphibian conservation.

## 1. Introduction

Many frogs have a peculiar mode of reproduction, such as laying eggs out of water in foam nests. This foam is composed of biomolecules released by the female, and the nest is built during amplexus with the aid of male frog’s leg movements [1,2,3]. Foam nests can be constructed on puddles, directly on the ground, on leaves, or at the soil–water interface [4]. Regardless of the anuran species, foam nests are mainly composed of proteins known as ranaspumins [5] and carbohydrates. Among these proteins are surfactants, uncommon lectins, as well as carbohydrates, which are likely to be responsible for the architecture and stability of the nest [3,5,6]. Although the functions of the chemical components of frog foam nests remain in the hypothetical field, it is noteworthy that many proteins analyzed so far have novel primary sequences and conformational structures, which make these biofoams a reservoir of new molecules [5,6].

In particular, frogs of the Leptodactylidae family in the Brazilian Atlantic Forest have the largest number of known reproductive modes (RM) (17 types), most of which involve the deposition of eggs in foam nests (9 types) either built in water bodies or on the ground [7,8]. For this family, such a variety of reproductive modes seems to have evolved as a strategy for the exploitation of microhabitats in the humid forests where they occur [1,8]. Foam nests protect eggs and embryos from direct contact with water from small, temporary, hot, and poorly oxygenated pools [1,9]; they provide defense against ultraviolet radiation, predators, and desiccation [3,6]; they increase the chances of egg fertilization [10]; and they serve as food reserves for tadpoles, which complete their development within the nests [11].

In addition to the chemical components, frog foam nests host a microbial community that has only recently begun to be fully characterized with the help of next-generation sequencing [12]. Studies have shown that in old-world rhacophorid frogs, the foam serves as a medium for vertical transmission of microbes to tadpoles, potentially playing a role in their healthy development [12]. The vital importance of foam nests for the reproduction, development, and health of the anurans reinforces the urgent need for environmental protective action. This is especially important given the increasing impact of anthropogenically driven environmental changes such as habitat destruction, chemical pollution, and climate change [13,14]. Furthermore, amphibian populations have been suffering constant threats due to epidermal infections caused by the fungus *Batrachochytrium dendrobatidis* (*Bd*), which has led to the extinction of 90 species and the decline in 501 populations [15].

Frog skin microbiota is well known to play an important role in its host’s health, and disturbances to its composition can increase the host’s susceptibility to pathogens [16]. This is also true for chytridiomycosis, as bacterial isolates from frog skin have been shown to inhibit the growth of *B. dendrobatidis* [17]. Despite the increasing focus on amphibian skin microbiome research, the role of the foam nest microbiome in shaping skin microbiota in species that develop entirely within foam nests remains unstudied.

In this study we characterize the foam nest microbiome of three neotropical frog species from the family Leptodactylidae, each representing a distinct mode of reproduction, as well as the microbiomes of associated environmental samples and host tissues. The goal is to identify the key factors influencing microbial community structure and composition. This knowledge is critical to understanding the impact of foam nest microbiomes on early and late host colonization and pathogen defense.

## 2. Materials and Methods

### 2.1. Sample Collection

Sample collection was performed during the rainy season, between January and March 2018, in two localities of the state of Ceará in northeastern Brazil, with proper regards to Brazilian regulations (license number 58036-2-SISBIO and AD025FF-SisGen released by national environmental agencies).

**Figure 1:**
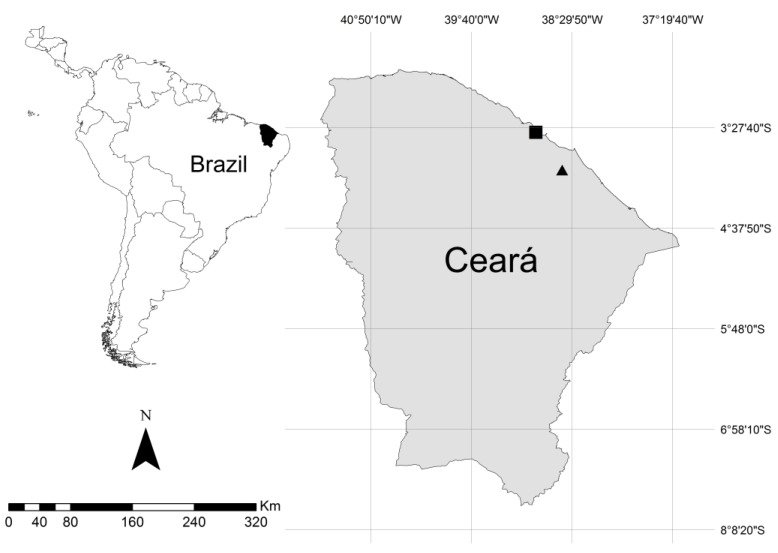
Geographical location of the collection sites; the square mark represents Fazenda Maceió-Taíba, and the triangle mark represents RPPN Monte Alegre.

Foam nests of the frogs *Leptodactylus vastus* (two foam nests) and *Physalaemus cuvieri* (three foam nests), and samples of their associated water (two samples), were collected in RPPN Monte Alegre, located in Serra da Aratanha between the municipalities of Maranguape and Pacatuba (03°57′10” S, 38°36′48” W). Three foam nests of the frog *Adenomera hylaedactyla*, and their respective samples of associated soil, were collected in Fazenda Maceió, located in Taiba in the municipality of São Gonçalo do Amarante (03°30′54.9” S, 38°55′07.7” W) (Figure 1). Figure S1 in the Supplementary Material shows photographs of the collected foam nests.

All samples were collected aseptically using sterile material. Foam nests of *L. vastus* and *P. cuvieri* were carefully collected from the water surface or from the edges of temporary standing water and placed in sterile flasks. Nests from *A. hylaedactyla* were gently removed from their soil cavities, avoiding cross contamination with plant debris and soil particles. Samples of water and soil were also placed in sterile tubes, kept in a cool box, and brought to the laboratory. Eggs, sand, leaves, and branches were manually removed from foam nests under aseptic conditions, and the samples were subsequently stored at −20 °C until further use.

This study also analyzed tissue samples of *L. vastus*, aiming to compare the origin and composition of the microbiomes. For this task, a female specimen was also collected in RPPN Monte Alegre and euthanized by intracephalic administration of 30 mg/kg of lidocaine hydrochloride 2%, according to international animal ethics standards of the American Veterinary Medical Association [18] with a permit from the Ethics Committee on Animal Use of the Federal University of Ceara (CEUA, license number 6200160418). Subsequently, three sections of intestine, three of cloaca, and two of skin were dissected, freeze-dried using liquid nitrogen, and stored at −20 °C until further use. The specimen used in this study was deposited in the herpetological collection of the Federal University of Ceará (Voucher number: CHUFC A8618).

### 2.2. Biochemical Characterization of the Foam Nests

#### 2.2.1. Protein and Carbohydrate Determination

Total protein levels were quantified using the Coomassie Blue method [19], using bovine serum albumin (BSA) as a standard. Total carbohydrate concentrations were determined using the sulfuric acid–UV method [20].

#### 2.2.2. Surface Tension

The surface tension of the foam was measured at room temperature via the Du Noüy ring method [21] using a Krüss K6 tensiometer (Kruss GmbH, Hamburg, Germany). As a negative control, the surface tension of the water was evaluated, obtaining about 71 mN/m. For statistical analysis, a one-way ANOVA with Tukey test was performed using GraphPad Prism 8.0.1 software (San Diego, CA, USA) [22].

#### 2.2.3. SDS-PAGE

The protein profile of the foam nests was revealed via tricine-SDS-PAGE [23]. The final concentration of the separating gel was 16,5% T, 3% C acrylamide/bis-acrylamide. A total of 5 μg of protein was applied in the gel. Molecular markers ranged from 10 to 225 kDa (Promega Corporation, São Paulo, Brazil). The gel was fixed in 50% ethanol/10% acetic acid/40% distilled water (*v*/*v*/*v*) for 1 h and washed for 10 min twice with distilled water. Next, it was stained using the colloidal Coomassie Brilliant Blue G-250 method [24]. Destaining was performed using acetic acid 1%.

### 2.3. DNA Extraction and Sequencing

DNA extraction was performed on three 0.5 g subsamples of each foam nest and three subsamples of the soil associated with the *A. hylaedactyla* foam nest using a DNeasy Power Lyzer Power Soil Kit (Qiagen, CA, USA), according to the manufacturer’s instructions. For the water samples associated with the nests of *P. cuvieri* and *L. vastus*, 50 mL of each sample was centrifuged, and the total DNA was extracted from the resulting pellet using the same protocol as for the foam nest and soil samples.

Regarding the tissue samples, intestine and cloaca were divided into three subsamples, and skin was divided into two subsamples. A total of 0.5 g of each subsample was incubated at 65 °C for 2 h in 750 µL of 20 mM of Tris-HCl pH 7.5, 100 mM EDTA, and 50 µL of Proteinase K. Then, 750 µL cetyltrimethylammonium bromide (CTAB) solution was added (2% CTAB 2%, 1,4 M NaCl, 20 mM EDTA, and 100 mM Tris-HCl pH 8.0) and incubated at 60 °C for 16 h. The CTAB protocol was based on the method described by Warner in 1996 [25]. The resulting DNA was resuspended in 50 µL Tris-HCl (10 mM; pH 8.0) containing 20 μg/μL of RNAse.

Concentrations and quality of all DNA subsamples were evaluated through absorbance measurements at 260 nm, 280 nm, and 230 nm using a Nanodrop^®^ ND-1000 spectrophotometer (NanoDrop, Wilmington, DE, USA).

The V4 region of the 16S bacterial rRNA gene was amplified by PCR using the primers 515F (5′-GTGCCAGCMGCCGCGGTAA-3′) and 806R (5′-GGACTACHVHHHTWTCTAAT-3′) [26]. The PCR reaction was performed in a final volume of 25 μL containing 20 ng of genomic DNA (template), 1X buffer solution containing 12 mM MgCl_2_, 0.3 mM of each dNTP, 0.3 μM of each primer, and 1.0 unit of platinum Taq polymerase high fidelity. A control reaction was performed by adding water instead of DNA. The PCR conditions were 94 °C for 4 min to denature the DNA, with 35 cycles at 94 °C for 45 s, 50 °C for 60 s, and 72 °C for 180 s, with a final extension at 72 °C for 10 min. After indexing, PCR products were purified using Agencourt AMPure XP-PCR beads (Beckman Coulter, Brea, CA, USA), following the manufacturer’s instructions. After quantification on a Qubit 2.0 fluorometer (Invitrogen, Carlsbad, CA, USA), different volumes of each library were pooled into a single tube such that each amplicon was represented equally. The pool was diluted to 4 nM, denatured, and further diluted to a final concentration of 10.0 pM with 20% PhiX (Illumina, San Diego, CA, USA). Sequencing was performed with the MiSeq Reagent Kit v2 (300 cycles, paired-end sequencing 2 × 150 bp) on the Illumina MiSeq platform at the Genomics and Bioinformatics Center (CEGENBIO/NPDM) of the Federal University of Ceara (UFC), Brazil.

### 2.4. Data Processing

The 43 datasets obtained in the sequencing of the nests (9 subsamples of *A. hylaedactyla*, 6 of *L. vastus,* and 9 of *P. cuvieri*), soil (9 subsamples), water (two subsamples), and frog tissues (3 subsamples of gut, 3 of cloaca, and two of skin) were analyzed using bioinformatics tools as follows.

Illumina adapter sequences were trimmed from the already demultiplexed raw FASTQ files using Cutadapt v1.8 in paired-end mode. Quality control of the reads was performed using FastQC v.0.11.8 [27] and vsearch v2.10.4 [28]. Subsequent analyses were performed within the R v3.5.3 environment [29], following the DADA2 v1.11.1 package [30] pipeline suggested by the authors and adjusting parameters to our data. It resulted in a table of non-chimeric amplicon sequence variants (ASVs) [31], which records the number of times each ASV (sequence differing by as little as one nucleotide) is observed in each sample. DADA2 identifies more real variants and outputs less spurious sequences than traditional operational taxonomic unit (OTU) clustering methods [30]. Taxonomy assignment and removal of non-bacterial sequences was performed against the SILVA database [32]. Samples were subsequently rarefied at 23,287 reads per sample to normalize read counts across samples. Samples that were outside of the rarefaction curve were eliminated, resulting in 20 foam nest datasets (8 subsamples of *A. hylaedactyla*, 5 of *L. vastus,* and 7 of *P. cuvieri*), comprising a total of 39 datasets.

### 2.5. Statistical Analysis

After rarefaction, a total of 20 foam nest subsamples were analyzed: 8 subsamples of *A. hylaedactyla*, 5 of *L. vastus,* and 7 of *P. cuvieri*. Alfa diversity estimators (Chao1, Shannon, and Inverse Simpson) were calculated, and we used Kruskal–Wallis tests for differences across host anuran species’ foam nests. To estimate how representative our foam nest samples were of the bacterial community, Good’s coverage estimator was calculated for all samples, as well as rarefaction curves. For beta diversity analysis, foam nest subsamples were clustered using an unweighted pair group method with an arithmetic mean (UPGMA), to determine clustering patterns across host species. UPGMA was used on Bray–Curtis distances of mean Hellinger-transformed ASV-relative abundances at the genus level. A UPGMA Bray–Curtis cluster was also made comparing the relative abundance of ASVs at the genus level of these foam nest microbial communities and those found in subsamples of their deposition environment (water and soil), parental tissues (*L. vastus* female gut, cloaca, and skin), and foam nests from three species of *Polypedates* belonging to the Rhacophoridae family in Borneo that were studied by McGrath-Blaser et al. [12]. Sequences were downloaded from the study by McGrath-Blaser et al. [12] under the access numbers SAMN18106736, SAMN18106737, SAMN18106738, SAMN18106756, SAMN18106757, SAMN18106758, SAMN18106776, SAMN18106775, and SAMN18106774 via BioProject ID PRJNA705959.

The diversity estimators, rarefaction curves, UPGMA Bray–Curtis heatmap and all statistics were completed using vegan package v2.5.4 [33] and an R statistical package [29]. All plots were generated using ggplot2 v3.2 [34].

## 3. Results

### 3.1. Biochemical Characterization of the Foam Nests

Protein and carbohydrate levels in foam nests varied among species, with *P. cuvieri* exhibiting the highest concentrations. The surface tension activity of surfactant proteins in the foam nests was found to be correlated with protein concentration, as demonstrated by the lower tension obtained in the sample from *P. cuvieri* (Table 1).

**Figure 2:**
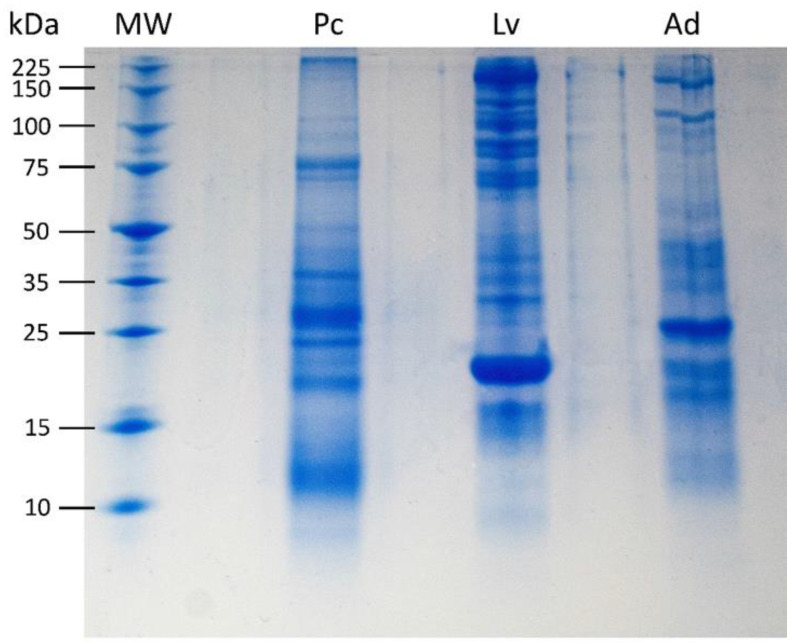
Tricine-SDS-PAGE of 5 μg foam nest fluid from *P. cuvieri* (Pc), *L. vastus* (Lv), and *A. hylaedactyla* (Ad). Molecular weight (MW) range of 10 to 225 kDa. The most intense band in Lv, of 23.5 kDa, corresponds to Lv-ranaspumin as described in Hissa et al., 2014 [35].

The electrophoresis gel revealed distinct protein profiles for each foam nest (Figure 2), with the foam nest of *L. vastus* exhibiting a greater apparent protein richness. The nests displayed only a few bands with the same molecular mass, indicating that the foam composition is unique and characteristic of each species. The foam nest of *L. vastus* displayed more distinctive protein bands, including an intense band at 23.5 kDa corresponding to Lv-ranaspumin [35,36]. Furthermore, *L. vastus* showed more prominent bands above 50 kDa compared to *P. cuvieri* and *A. hylaedactyla*.

### 3.2. Estimation of Bacterial Richness and Diversity in Foam Nests

**Figure 3:**
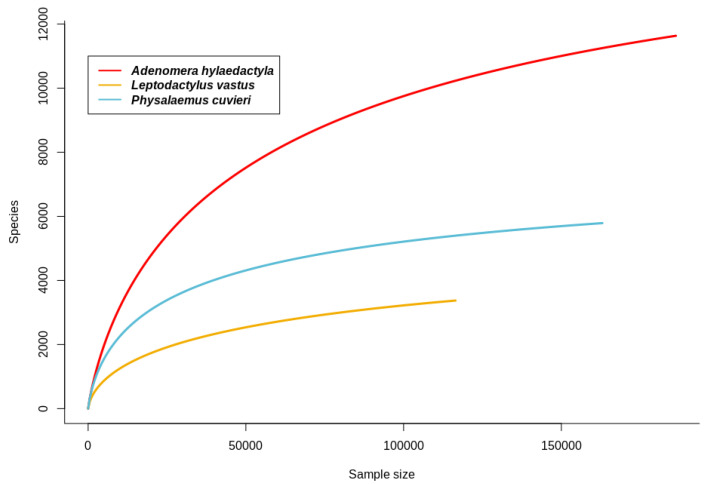
Rarefaction curves for observed amplicon sequence variants (ASVs) in foam nests from neotropical frogs *A. hylaedactyla*, *L. vastus*, and *P. cuvieri*.

A total of 20 foam nest 16S rRNA libraries were sequenced from the studied frog species: 8 from *A. hylaedactyla*, 5 from *L. vastus,* and 7 from *P. cuvieri*. After filtering out low-quality and short-sequence reads, a total of 774,363 raw sequences were obtained, with an average of 38,718 sequences/sample (ranging from a maximum of 86,865 to a minimum of 26,711). The largest variation was observed in the samples of *A. hylaedactyla*, ranging from a maximum of 86,865 to a minimum of 26,711, with an average of 42,209. The samples from *L. vastus* showed less variation (ranging from a maximum of 67,934 to a minimum of 23,287, with an average of 45,246), as did the samples of *P. cuvieri* (ranging from a maximum of 39,044 to a minimum of 23,433, with an average of 30,066). The rarefaction curves for the observed amplicon sequence variants (ASVs) approached the asymptotes, indicating that the sequencing and sampling efforts adequately captured the taxonomic diversity within each sample (Figure 3).

The richness of *A. hylaedactyla* was found to be higher and more statistically significant as compared to the other two species, as determined by both the Kruskal–Wallis test for observed richness (*p* = 0.001) and Chao1 (*p* = 0.001) (Table 2). No difference was observed between *L. vastus* and *P. cuvieri* (Kruskal–Wallis, observed richness *p* = 0.372 and Chao1 *p* = 0.371). The Shannon index was highest for *A. hylaedactyla* samples, followed by *P. cuvieri* and *L. vastus*, with a significant difference among the three species (Kruskal–Wallis, *p* = 0.019). However, the distinction did not occur between *A. hylaedactyla* and *P. cuvieri* (Kruskal–Wallis, *p* = 0.418) or *P. cuvieri* and *L. vastus* (Kruskal–Wallis, *p* = 0.088), with a significant difference only between the values of *A. hylaedactyla* and *L. vastus* (Kruskal–Wallis, *p* = 0.003). For the inverse Simpson index, *A. hylaedactyla* and *L. vastus* showed similar values (Kruskal–Wallis, *p* = 0.29), indicating a higher dominance level compared to *P. cuvieri*, which showed a value significantly higher than the other two species (Kruskal–Wallis, *p* = 0.05).

### 3.3. Microbial Community Structure and Composition

**Figure 4:**
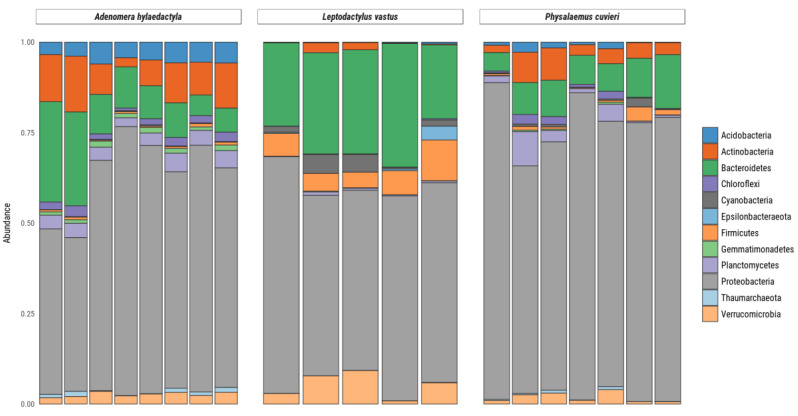
Relative abundance of bacteria and archaea amplicon sequence variants at phylum levels present in frog foam nests. Stacked bar plot of the relative abundance above 1%.

High-resolution community profiles were generated by processing reads using a denoised pipeline to resolve 16S rRNA gene ASVs at the single-nucleotide level. Bacterial sequences were predominant, accounting for 29.159 sequences, while only 116 sequences were identified as archaea. Out of the 12 phyla identified in the samples, those with an abundance above 1% were Acidobacteria, Actinobacteria, Bacteroidetes, Chloroflexi, Cyanobacteria, Epsilonbacteraeota, Firmicutes, Gemmatimonadetes, Planctomycetes, Proteobacteria, Thaumarchaeota and Verrucomicrobia (Figure 4). Among these, Proteobacteria were the most abundant, present in all samples at levels above 50%, followed by Bacteroidetes, Firmicutes and Actinobacteria. The other phyla comprised <5% of the total.

In *A. hylaedactyla,* the most frequent phyla were Proteobacteria (60.3%), with the majority belonging to the class Gamma-proteobacteria (56.6%) and a small proportion belonging to Alpha-proteobacteria (3.7%). The next most abundant phyla were Bacteroidetes (11%) and Actinobacteria (6.1%). In *L. vastus*, there was a predominance of Proteobacteria (58.2%), including Gamma-proteobacteria (42.9%) and Alpha-proteobacteria (15.3%). The next most abundant phyla were Bacteroidetes (26.9%) and Firmicutes (7.3%). The nests of *P. cuvieri* were predominantly composed of Proteobacteria (72.8%), primarily from the Gamma-proteobacteria class (71.2%) with a small contribution from Alpha-proteobacteria (2.0%) and Bacteroidetes (8.5%).

**Figure 5:**
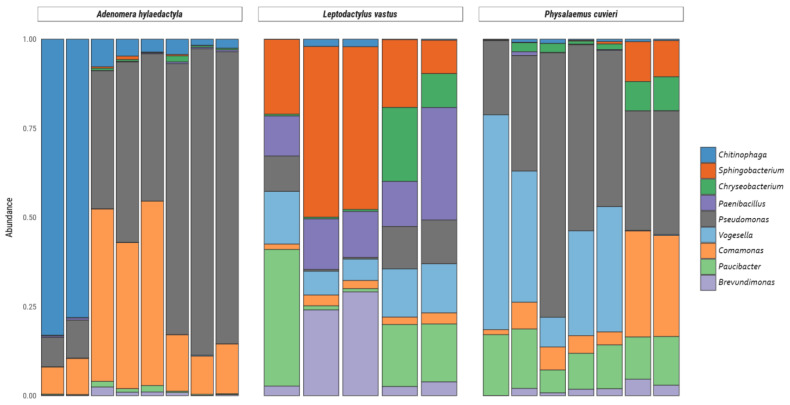
Relative abundance of bacteria and archaea amplicon sequence variants at the genus level present in frog foam nests. Stacked bar plot of the relative abundance above 10%.

It is noteworthy that some bacterial genera such as *Pseudomonas*, *Vogesella*, *Chryseobacterium*, *Chininophaga*, *Paenibacillus*, *Comamonas*, *Paucibacter*, *Brevundimonas,* and *Sphingobacterium* were the most abundant genera in the nests of the three frog species, comprising more than 10% of all bacterial taxa observed (Figure 5).

Among the Proteobacteria phylum, the genus *Pseudomonas* accounted for 49.2% of the diversity in *A. hylaedactyla*, 41.7% in *P. cuvieri,* and only 7.0% in *L. vastus*. Likewise, the genus *Comamonas* showed higher mean frequencies in *A. hylaedactyla* (24.9%) and *P. cuvieri* (11.7%) compared to *L. vastus* (2.4%). The genus *Vogesella* was more prevalent in the nests of *P. cuvieri* (24.3%) and *L. vastus* (11.0%) compared to *A. hylaedactyla* (<1.0%). Similarly, the genus *Paucibacter* was more abundant in the nests of *L. vastus* (14.8%) and *P. cuvieri* (12.6%), with minimal representation in *A. hylaedactyla* (<1%). The genus *Brevundimonas* was only representative in *L. vastus* nests (12.5%).

In the phylum Bacterioidetes, the genus *Chitinophaga* was predominantly present in the nests of *A. hylaedactyla*, with a mean frequency of 23.2%, while it had minimal representation in the other foam nests microbiomes (<1%). The genus *Sphingobacterium* was significant only in the nests of *L. vastus*, with a mean frequency of 28.6%. The genus *Chryseobacterium* presented low frequencies, with significant presence only in the nests of *L. vastus* (6.4%) and *P. cuvieri* (3.6%). The phylum *Firmicutes* showed a significant presence of the genus *Paenibacillus* only in the nests of *L. vastus*, with a mean frequency of 16.5%.

### 3.4. Beta Diversity of Foam Nest Bacterial Community

**Figure 6:**
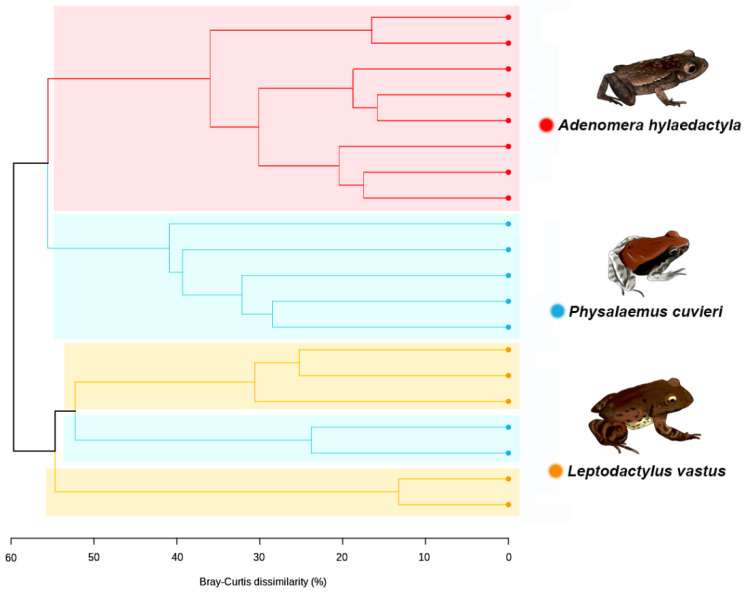
Dendrogram generated via UPGMA clustering analysis using the amplicon sequence variants (ASVs) at bacterial genus level, showing the relationship among the frog species.

To assess the bacterial beta diversity, the unweighted pair-group method with arithmetic mean (UPGMA) was carried out using the Bray–Curtis algorithm. The differences among the samples were further confirmed via clustering analysis. The analysis showed that the bacterial genus level ASVs were grouped into two main clusters (Figure 6). One cluster subdivided into two clear subgroups, one containing all replicates of *A. hylaedactyla* and the other containing most replicates of *P. cuvieri*. The second main cluster subdivided in two subgroups, one consisting of three *L. vastus* replicates and two *P. cuvieri* replicates, indicating similarity between them, while the other group only consisted of *L. vastus* replicates.

**Figure 7:**
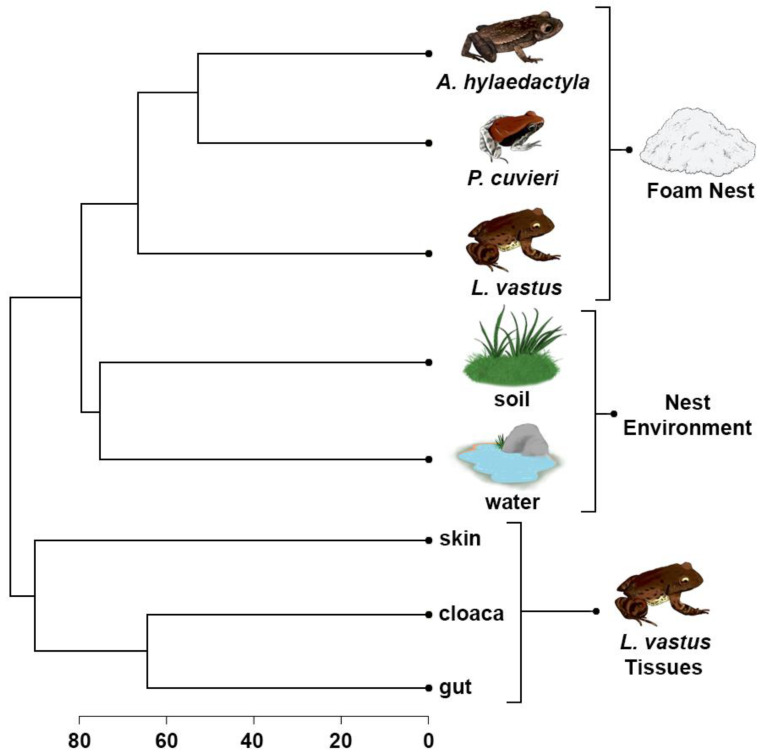
UPGMA cluster based on Bray–Curtis dissimilarities between bacterial amplicon sequence variants at the genus level presents in the foam nests, nest environments, and tissues of *L. vastus*.

The UPGMA dendrogram was used to access the differences between foam nests, nest environments, and host tissues to understand their relationships. The analysis revealed two main clusters, the first of which was divided into two subgroups, one containing foam nests and the other containing environmental samples. The second major group consisted solely of *L. vastus* tissue samples, with skin grouped separately from gut and cloaca microbiomes (Figure 7).

These results confirm the closer similarity between the foam nest microbiomes of *A. hylaedactyla* and *P. cuvieri*, despite the former laying eggs on land and the latter in water. Additionally, the closest species, *L. vastus* and *A. hylaedactyla*, were grouped separately, suggesting that microbiome composition is not driven by phylogeny. Although host-associated microbes are presumably acquired from the environment, the composition of the foam nest microbiome is distinct from that of free-living microbial communities. We performed an in-silico comparative analysis that included microbiome samples from three old-world rhacophorid species, all of which build their foam nests in trees—RM33 [7]. This analysis aimed to further investigate the influence of chemical composition on the structure of the microbial community (Figure S2, Supplementary Material). The results showed that, regardless of the reproductive mode (aquatic, terrestrial, riparian, and arboreal) or phylogenetic relationship (Leptodactylidae versus Rhacophoridae), the foam nests grouped together in a common cluster that was distinct from all other analyzed microbial communities. The unique chemical composition of frog foam nests supports this hypothesis.

## 4. Discussion

### 4.1. Biochemical Characterization of the Foam Nests

Despite the biological relevance of foam nests for frog development and evolutionary success, our understanding of their chemical composition is limited to a few species [3,37,38]. However, we do know that foam nests are rich in novel surfactant proteins [6,35], along with other proteins, lectins, and carbohydrates [3,5,38]. The microbiome of frog foam nests is even less well understood, and we are just beginning to learn about their composition and role on early host colonization [12]. The effects of variation in nest size and foam consistency between species on microbiome composition are still unknown.

Biochemical analysis of fluid from foam nests of *A. hylaedactyla*, *P. cuvieri,* and *L. vastus*, which spawn on land, water, and land–water interface (riparian), respectively, confirmed the presence of carbohydrates and protein in varying proportions (Table 1). Electrophoresis gel analysis revealed that each nest has its own set of proteins and that protein richness varies among species, with *L. vastus* having the most protein bands and *A. hylaedactyla* the fewest. A strong protein band stands out in each sample. In *L. vastus*, the apparent 23.5 kDa band corresponds to Lv-Rsn-1, its main surfactant protein [35]. Although electrophoresis is not robust enough to reveal the proteome of the foam nests, it did show that protein composition is species-specific (Figure 2). Thus, we suggest that the proteome plays a role not only in nest building and stability [6], but also in the selection and establishment of the nest microbiome.

According to Fleming at al. [6], initial foam formation involves a specific surfactant protein (Rsn-2) establishing hydrophobic interactions with lectins (Rsn-3 to Rsn-6), which then bind to carbohydrates to give rise to the nest architecture. This model, established for the fluid secreted by the túngara frog (*Engystomops pustulosus*), explains the stability of the nest during the tadpole’s development until metamorphosis, when the nest disintegrates. Whether this model applies to other species remains to be proven. So far, nine ranaspumins have been isolated from frog foam nests, but only two of them, Rsn-2 from *Engystomops pustulosus* [39] and Lv-Rsn-1 from *L. vastus* [35], have been studied due to their surfactant activity. Despite this, these two proteins have different molecular weights, amino acid sequences, and 3D structures [35,39]. Furthermore, we have not yet been able to demonstrate the occurrence of lectins in the foam nest of *L. vastus* (unpublished data) that supports a similar model of foam nest stabilization.

### 4.2. A distinctive Frog foam Nest Community

Our study provides new insights into the composition and structure of the microbiome in frog foam nests. The results of our analyses showed that the richness and alpha diversity of nests are not linked to frog phylogeny, since closer species presented significantly different results (Table 2). Although the three species belong to the Leptodactylidae family, *L. vastus* and *A. hylaedactyla* are closely related, belonging to the Leptodactylinae subfamily, whereas *P. cuvieri* belongs to the Leiuperinae subfamily (sensu [40,41]). Our findings also suggest that nest size and environment influence ASV richness and diversity. The smaller foam nest of *A. hylaedactyla*, which is laid on land and measures about 2.5 cm, had significantly higher bacterial richness and diversity compared to the larger aquatic and riparian nests of *P. cuvieri* (5.0 cm) and *L. vastus* (15.0 cm). This could be because smaller nests have a greater surface area in contact with the environment, and terrestrial environments in general have higher bacterial richness than non-marine aquatic environments [42]. Similarly, amphibian skin microbiomes tend to be richer in species with terrestrial habits, whereas aquatic and arboreal species have lower richness values [43,44].

**Figure 8:**
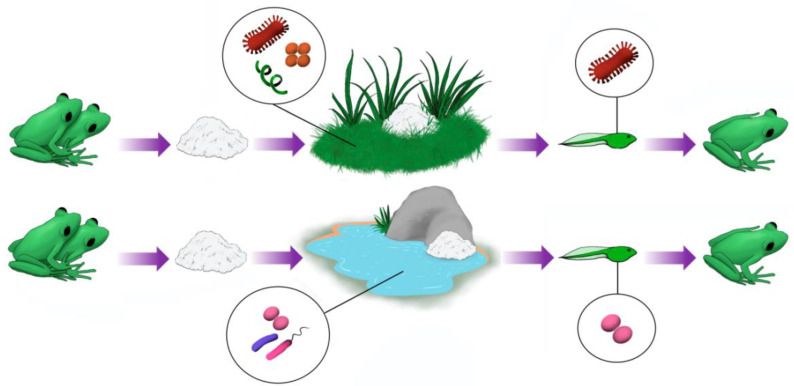
Anuran foam nest microbiomes originate from the microbiome of the environment where these nests are deposited. However, certain groups of microorganisms are clearly enriched in nests compared to their natural environments. This suggests that these microorganisms may be beneficial to tadpoles in the early stages of development.

Our study showed that beta diversity in foam nests is clearly different from that found in the other groups herein studied (Figure 8). The main driver of the clustering of the foam nest assemblage was the dominance of Proteobacteria (between 58% for Gamma-proteobacteria and 73% for Alpha-proteobacteria), while the water and soil also presented a high amount of Proteobacteria (approximately 50% for water and 25% for soil) together with Actinobacteria (approximately 11% for water and 17% for soil) and Acidobacteria (approximately 6% for water and 14% for soil).

*Leptodactylus vastus* gut and cloaca presented higher amounts of Firmicutes (between 17 and 29%), followed by Bacteroidetes (between 12 and 24%). Skin samples, together with foam nests, presented a higher abundance of Proteobacteria (between 46 and 59%), followed by Bacteroidetes (between 4 and 10%) and Firmicutes (between 4 and 5%); however, other phyla composed less than 2% in skin samples.

Members of Proteobacteria and Bacteroidetes taxa have been shown to dominate the bacterial community of the rhacophorid frogs’ foam nests from Borneo and the skin microbiomes from several anuran species [12]. They also influence the secretion of volatile compounds of the South American tree frog *Boana prasina* [45], production of antifungal molecules [43,46], and/or antimicrobial peptides [47]. The phylum Firmicutes has similarly been reported as frequent in amphibian skin microbiomes [43,44,47,48].

The studied foam nests had low representation of the phyla Acidobacteria, Actinobacteria, Chloroflexi, Cyanobacteria, Epsilonbacteraeota, Gemmatimonadetes, Planctomycetes, Thaumarchaeota, and Verrucomicrobia, which are also found at low frequencies in the digestive tracts and skin microbiomes of other amphibian species [44,49]. Although not very expressive in foam nest microbiomes, the phylum Actinobacteria was found with high frequency in the skin of the frog *Rhinella horribilis* in Costa Rica [48] and is dominant in adult *Anaxyrus boreas*, replacing Proteobacteria that is dominant in tadpoles [50]. Experiments have also shown a significant increase in the frequency of Actinobacteria and Planctomyces in the digestive tract microbiomes of tadpoles exposed to higher temperatures [51].

The bacterial genus *Pseudomonas* was found to be one of the most abundant in the foam nests studied, being especially abundant in *A. hylaedactyla*. *Pseudomonas* species are known as both environmental and host-specific symbionts and are commonly found in the skin microbiome of amphibians, where they are reported to play an important role [43,44,45,47,48,52,53,54,55,56,57,58,59,60]. *Pseudomonas* strains isolated from amphibians have demonstrated great potential against pathogenic microorganisms, including the panzootic *Batrachochytrium dendrobatidis* (*Bd*) and the human pathogen *Aspergillus fumigatus* [47,52,56,57]. This highlights the significance of *Pseudomonas* in amphibian defense and its potential for biotechnological applications, such as the isolation of pharmacological molecules. Notably, as skin symbionts, *Pseudomonas* strains were recently reported to be involved in the production of volatile sex pheromones in the frog *Boana prasina* [45,61].

The genera *Vogesella* and *Paucibacter* most frequently found in the foam nests of *P. cuvieri* and *L. vastus*, deposited in an aquatic environment, are mainly found in freshwater sources in temperate climates [62,63,64,65,66,67], pointing out an environmental contribution to the foam nest microbiome. Their ecological role is poorly studied, but it is known that *Vogesella mureinivorans* is capable of degrading the polysaccharides chitin and peptidoglycan [63]. The presence of carbohydrates in foam nests may contribute to colonization by *Vogesella* [3].

The genera *Chitinophaga*, *Chryseobacterium*, and *Sphingobacterium* are common in several environments or in symbiosis with several groups of organisms; however, *Chryseobacterium* is also a pathogen associated with several diseases, being commonly found in anura skin related to infections in the group [68]. On the other hand, *Chitinophaga* and *Sphingobacterium* are commonly found in environmental samples of soil and water, also related to fungi and plants endosymbionts [69,70,71,72,73]. Loudon et al. [54] showed that a *Chitinophaga arvensicola* isolate, together with a *Bacillus* sp., had a higher inhibitory effect against *Bd* compared to the *Bacillus* sp. alone.

*Paenibacillus* was shown to colonize the intestine of *Lithobates pipiens* tadpoles [74] and the salamander *Plethodon cinereus* [58]. This genus is noteworthy for its production of antifungal molecules.

McGrath-Blaser et al. have already demonstrated the presence of an exclusive bacterial community that colonizes frog foam nests [12]. These foam nests, produced by rhacophorid frogs, present a unique bacterial structure distinct from the environment, skin of tadpoles, and skin and cloaca of adult frogs. Although the structure of the foam nests is unique, some components of this community have already been reported as originating from the environment or the skin and digestive tract of adult frogs, suggesting that foam nests acquire their microbial communities from these sources.

The rhacophorid frogs are arboreal and lay their eggs in foam nests on tree leaves close to water, where tadpoles can complete their development [1,11,12]. While this study provides evidence of vertical transfer of the foam nest microbiome [12], these observations are preliminary and may not account for the diversity of foam nest microbiomes in other rhacophorid species or in other families that lay eggs in foam nests. This type of egg deposition evolved independently (convergently) in different anuran families with distinctive behaviors compared to arboreal rhacophorids [1,11,75].

Our findings indicate that the chemical composition of foam nests varies between the studied frog species, with each nest having a unique protein profile. Foam nests typically consist of 85–65% proteins and 35–15% sugars, many of which have been identified as novel proteins [3,6]. Currently, we have only limited knowledge of the foam nest chemical composition from two species of Leptodactylidae, *Leptodactylus vastus* [3] and *Egystomops pustulosus* [6], and one species of Rhacophoridae *(Polypedates leucomystax)* [76]. The major known protein in each nest, Lv-RSN-1 (from *L. vastus*) [3,35,36], RSN-2 (from *E. pustulosus* [39], and Ranasmurfin (from *P. leucomystax* [76], does not significantly share amino acid sequence or conformational structure. These novel proteins likely play a crucial role in nest architecture and stability [35,37] and, together with other well-known defense proteins detected in foam nests, represent an efficient protection strategy for the development of tadpoles (3, 36, 38]. We have recently analyzed the proteome of the foam nests of *L. vastus*, *L. macrosternum*, *P. cuvieri* and *P. albifrons,* which revealed a high number of unidentified protein spectra as well as a significant number of species-exclusive spectra, ranging from 75% exclusive to *L. vastus* to 55% exclusive to *P. cuvieri* (unpublished data), suggesting a diverse range of proteins across different species. However, many of these unidentified proteins are due to the lack of annotated genomes of foam nest-producing frogs, which is further highlighted by the fact that there are only 25 anuran genomes available, none of which correspond to species from Brazil or foam nest-producing species (www.ncbi.nlm.nih.gov, accessed on 25 November 2022). This underscores the need for more research to fully understand the relationship between the foam nest proteome and microbiome.

The exact relevance of the foam nest microbiome to host development, immunity, and nutrition is yet to be determined, but our results suggest a key functional role, considering that distinct frog species select their own microbiomes, despite sharing some common taxa across different environments. However, due to the vulnerability of foam nests to anthropic pressure, there is a pressing need for conservation efforts to protect amphibian species that reproduce by depositing eggs in foam nests.

## 5. Conclusions

This study provides significant evidence that neotropical frogs of the Letptodactylidae family acquire their foam nest microbiota from the environment, with the unique chemical composition of the foam nest being a more important driver of the microbiome than phylogeny and the environment of nest deposition. Despite the diversity of reproductive modes among Letptodactylidae, the foam nest microbiomes share a core of phyla including Acidobacteria, Actinobacteria, Bacteroidetes, Chloroflexi, Cyanobacteria, Epsilonbacteraeota, Firmicutes, Gemmatimonadetes, Planctomycetes, Proteobacteria, Thaumarchaeota, and Verrucomicrobia, suggesting convergent evolution. The enrichment of certain taxa in foam nests, such as *Pseudomonas*, which is a key player in the anura skin microbiome, highlights the host’s early selection of beneficial microbes for defense against pathogens, such as the fungus *Batrachochytrium dendrobatidis*.

## Figures and Tables

**Table 1:** Concentration of proteins and carbohydrates and surface tension activity in foam nests of neotropical frogs that have different modes of reproduction.

Species	Protein (mg/mL)	Carbohydrate (mg/mL)	Surface Tension (mN/m)	Reproductive Mode ^†^
*P. cuvieri*	2.27 ± 0.48 ^a^	1.16 ± 0.06 ^a^	39.66 ± 0.50 ^a^	RM 11
*L. vastus*	1.37 ± 0.24 ^b^	0.23 ± 0.01 ^b^	45.34 ± 0.70 ^b^	RM 13
*A. hylaedactyla*	0.75 ± 0.15 ^b^	N/A	50.73 ± 0.35 ^c^	RM 32

**^†^** Amphibian reproductive modes based on Haddad and Prado, 2005 [7]. **^N/A^**—Not analyzed. Different letters in each column represent significant differences at *p* < 0.05.

**Table 2:** Richness and alpha diversity of amplicon sequence variants (ASVs) in the foam nests of *A. hylaedactyla*, *P. cuvieri*, and *L. vastus*.

Richness/α-Diversity Index	Leptodactylidae Species		
	*A. hylaedactyla*	*P. cuvieri*	*L. vastus*
ASVs	**3555.38 ± 167.89**	1706.43 ± 307.93	1198.60 ± 159.07
Chao1	**4631.60 ± 316.39**	1977.25 ± 355.38	1544.21 ± 219.95
Shannon	**6.21 ± 0.15**	5.93 ± 0.31	4.95 ± 0.20
Inverse Simpson	57.83 ± 11.48	**165.86 ± 56.58**	40.89 6.89

## Data Availability

Data are available from corresponding authors under reasonable request.

## Supplementary Materials

The following supporting information can be downloaded at: https://www.mdpi.com/article/10.3390/microorganisms11040900/s1.
