# Supplementary material for: Neotropical Frog Foam Nest’s Microbiomes
Source: Microorganisms. 2023 Mar 30;11(4):900. doi: 10.3390/microorganisms11040900 (PMC10146838; doi:10.3390/microorganisms11040900)
Supplement: Supplementary file 1 [file microorganisms-11-00900-s001.zip › microorganisms-2256876-supplementary.pdf]

Supplementary Material

Chemical composition and reproductive modes drive the microbial acquisitions of Neotropical frog foam nest microbiome

Felipe Augusto Correia Monteiro, Saulo Gonçalves de Santiago Bezerra, Luzia Gabrielle Zeferino de Castro, Francisca Andrea da Silva Oliveira, Leonardo R.O. Normando, Vânia Maria Maciel Melo and Denise Cavalcante Hissa

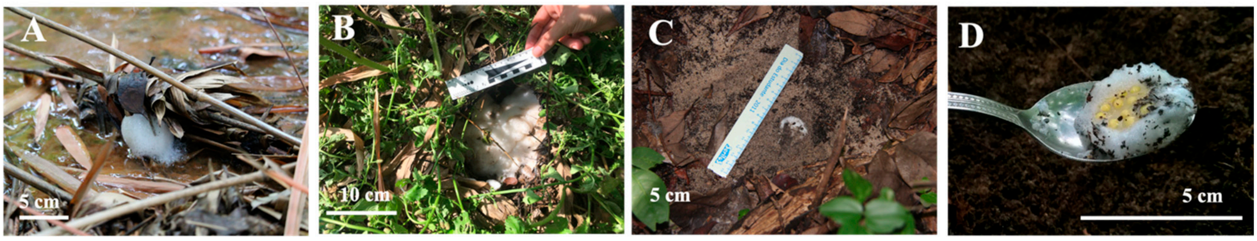

Figure S1. Foam nest of *Physalaemus cuvieri* (A), *Leptodactylus vastus* (B) and *Adenomera hylaedactyla* (C,D). Figure D shows the foam nest after excavated from ground.

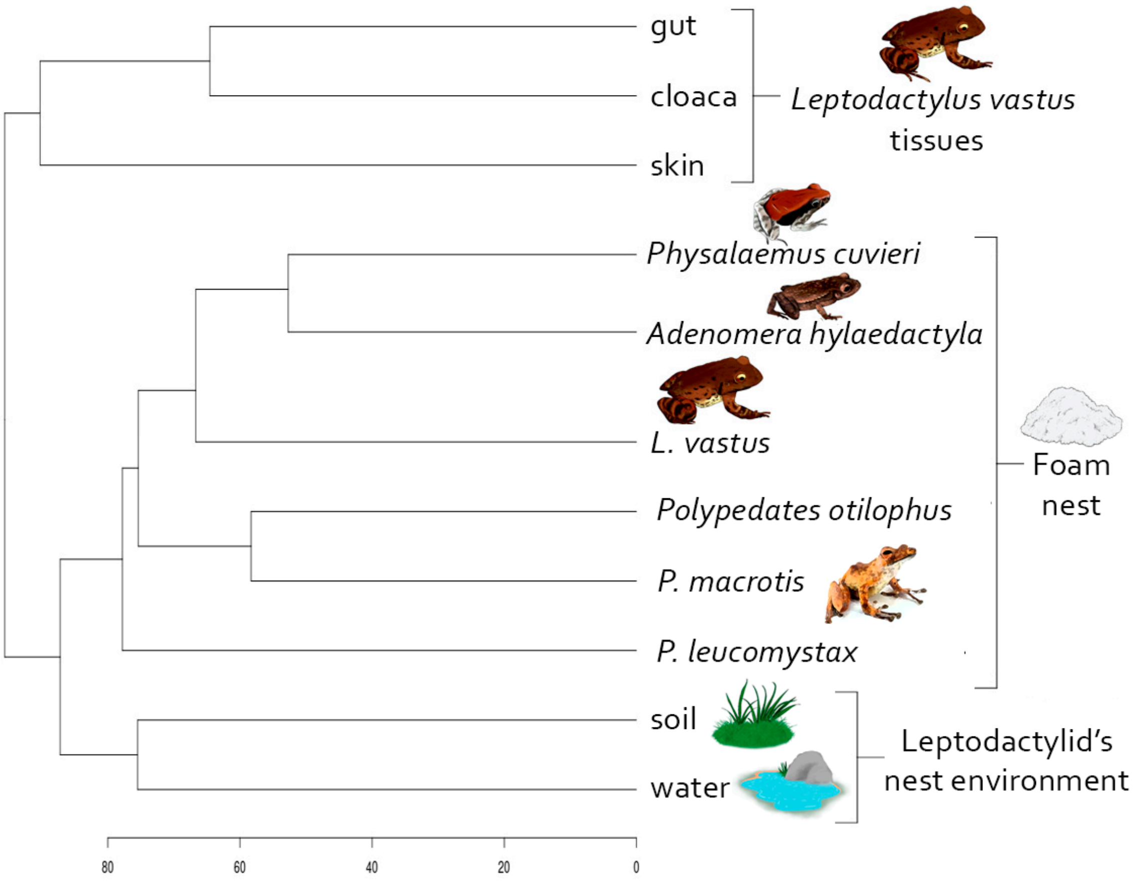

Figure S2. UPGMA cluster based on Bray–Curtis dissimilarities between bacterial amplicon sequence variants at genus level presents in the Leptodactylidae and Rhacophoridae foam nests, leptodactylid's nest environments and tissues of *L. vastus*. Sequences from Rhacophoridae foam nests were taken from the study of McGrath-Blaser et al. (2021) under the access number (SAMN18106736, SAMN18106737, SAMN18106738, SAMN18106756, SAMN18106757, SAMN18106758, SAMN18106776, SAMN18106775 and SAMN18106774) via BioProject ID PRJNA705959.
